# Occupational physicians’ perceptions of professional role change following health policy reform in France: A qualitative study

**DOI:** 10.1016/j.puhip.2026.100872

**Published:** 2026-10-10

**Authors:** Babacarsy Diop, Etienne Fourreau, Bénédicte Clin, Mathilde Boulanger

**Affiliations:** a CHU Caen, Service de Santé au Travail et Pathologie Professionnelle, Caen, France; b Université de Caen Normandie, Caen, France; c ASTER – Recherches en Santé au Travail, France; d INSERM U1086 « ANTICIPE », Caen, F-14000, France; e MIST Normandie, Hérouville-Saint-Clair, France

**Keywords:** Occupational health, Health Promotion, Public health policy, Workplace health, Qualitative Research, Occupational physicians

## Abstract

**Objectives:**

To explore occupational physicians’ perceptions of the 2 August 2021 reform and changes in their professional role, identify reported barriers and perceived facilitators to practice change, and describe reported health promotion practices.

**Study design:**

Qualitative study using semi-structured interviews.

**Methods:**

Ten occupational physicians practising in Normandy, France, were interviewed between November 2024 and March 2025. Data were analysed using thematic content analysis with a hybrid deductive–inductive coding approach.

**Results:**

Participants expressed contrasting perceptions of the reform, ranging from support for its objectives to concerns about feasibility and dilution of the occupational physician's specific role. Reported barriers were mainly organisational, including limited time, workload and staffing constraints. Dedicated time, additional resources, training and multidisciplinary support were described as conditions that could facilitate implementation. Participants also reported health promotion practices that predated the reform and were not always perceived as distinct public health activities.

**Conclusions:**

Among the physicians interviewed, few changes in practice were directly attributed to the reform, while several health promotion activities were reported to have predated it. The findings suggest a tension between expanded public health expectations and perceived boundaries of the occupational physician's role within an already constrained organisational context.

## 1 Introduction

Health systems are regularly shaped by reforms that expand professional roles and responsibilities. However, regulatory requirements and professional guidelines do not necessarily lead to sustained changes in practice, which also depend on individual and organisational factors such as professionals’ knowledge and perceptions, workload, available time and resources. [1], [2], [3], [4] Medical education may contribute to professional competencies and practice patterns, but training alone may not overcome organisational barriers to implementation. [1], [2], [5], [6]

Changes in professional missions may also raise questions regarding professional expectations and role boundaries. Previous studies in occupational medicine have shown variation in how occupational health functions and professional competencies are prioritised. [7], [8], [9] Qualitative research is well suited to exploring professionals’ perceptions and experiences within their context of practice. [10]

In France, the 2021 reform forms part of a longer evolution of occupational health. Over previous decades, successive reforms had already changed the organisation and missions of occupational medicine, notably by strengthening prevention and workplace interventions. These changes occurred in a context of demographic constraints, with a declining number of occupational physicians alongside a progressive expansion of their missions. In 2019, approximately 4000 occupational physicians were practising for more than 25 million workers. The 2021 reform should therefore be considered in the context of professional and demographic changes that preceded it. [11], [12], [13]

The French Law of 2 August 2021 aimed at strengthening prevention in occupational health explicitly expanded the missions of occupational health services. It incorporated into the French Labour Code their contribution to public health objectives and participation in workplace health promotion, including vaccination and screening campaigns, raising awareness of the benefits of physical activity and providing information on disability in the workplace. These provisions came into force on 31 March 2022 and formalised a more explicit relationship between occupational health, health promotion and public health objectives. [14]

How occupational physicians perceive these expanded missions and translate them into practice remains poorly documented. This qualitative study therefore aimed to explore occupational physicians’ perceptions of the 2 August 2021 reform and changes in their professional role, identify reported barriers and perceived facilitators to practice change, and describe reported health promotion practices.

## 2 Methods

### 2.1 Study design and participants

A qualitative study based on individual semi-structured interviews was conducted with occupational physicians practising in Normandy, France. Occupational medicine residents and collaborating physicians still in training were not eligible, as the study focused on physicians practising as occupational physicians.

For pragmatic reasons of feasibility and access, initial recruitment was conducted within the occupational health service where the researcher was undertaking his placement. Participants were invited through a collective invitation drafted by the researcher and distributed through the service's mailing list to all 22 occupational physicians working within the service at that time. Interested physicians contacted the researcher directly. Nine participants were recruited through this process. Most non-participants did not respond; one explicitly declined because of limited availability. A tenth occupational physician practising outside this service was subsequently identified through a professional contact.

Reporting of this study was informed by the Consolidated Criteria for Reporting Qualitative Research (COREQ) checklist. [15]

### 2.2 Researcher characteristics and reflexivity

All interviews were conducted by the first author, then an occupational medicine resident in his fifth semester of training, conducting his first qualitative study. Study preparation included prior literature and methodological work, and the interview guide, data collection and analysis were regularly discussed with the research supervisor.

Nine participants worked in the occupational health service where the researcher was undertaking his placement, across several geographically distinct centres. Pre-existing relationships with participants were professional in nature and varied in degree. Before recruitment, the researcher had not discussed the study topic or participants' views on the reform with any of the participants. To the researcher's knowledge, no specific service-wide meeting or collective work focusing on the public health missions introduced or reinforced by the reform had taken place before data collection. The tenth participant worked outside this service. The researcher had no personal relationships with participants.

The researcher approached the interviews without a predefined hypothesis regarding the effects of the reform and sought to maintain an open, non-evaluative approach allowing divergent views to be expressed.

### 2.3 Data collection

The semi-structured interview guide was developed from the study objectives, prior literature and documentary review, and the content of the 2 August 2021 reform, then discussed and validated with the research supervisor. No specific qualitative theoretical framework was used. The guide comprised 27 questions in five sections: participant and professional characteristics; perceptions of the reform; barriers and facilitators; training and support; and practices related to missions introduced or reinforced by the reform. Reformulations and probing questions were used when necessary. No formal pilot interview was documented. The full guide is provided as supplementary material.

Ten interviews were conducted between November 2024 and March 2025. Three took place face-to-face in participants' offices and seven by telephone, lasting 30–70 min. The decision not to audio-record interviews was made at the study design stage to minimise potential inhibition associated with recording when discussing participants' professional perceptions and practices. Methodological literature has highlighted that recording devices may influence interview interactions in some contexts. [16] Responses were typed in Microsoft Word by the researcher during each interview, aiming to preserve participants' wording as closely as possible. Participants could be asked to repeat, clarify or rephrase statements to allow immediate verification of the researcher's understanding and notes. Immediately afterwards, notes were reviewed, organised and prepared for analysis. They were not subsequently checked by participants, and no separate field notes were kept.

### 2.4 Data analysis

Data were analysed manually using thematic content analysis concurrently with data collection. [17] After each interview, data were progressively incorporated into a cumulative document organised by theme while retaining interview chronology. A coding table then grouped excerpts into themes, subthemes and ideas. A summary of the coding framework is provided as Supplementary Material 2.

Coding followed a hybrid deductive–inductive approach. Main analytical categories and some subthemes were defined as a priori from the study objectives and interview-guide domains, while unanticipated subthemes and ideas emerging from the data could be added during analysis. Of the five interview-guide sections, the first described participant and professional characteristics; the remaining four constituted the main analytical domains.

Initial coding was conducted by the first author. The coding table was repeatedly reviewed in detail with the research supervisor. Discussions prompted further exploration of some accounts, refinement of categories and, where interpretations differed, reconsideration of the classification of some data. The full dataset was not independently double-coded, and no specialised qualitative analysis software was used.

Thematic saturation was assessed through concurrent analysis. From the seventh interview onwards, no new major theme or analytical category requiring modification of the coding framework was identified; the subsequent three interviews mainly enriched, refined or illustrated existing categories. This was considered sufficient in relation to the study objectives.

Quotations were translated from French into English for the manuscript.

### 2.5 Ethical considerations

The institutional ethics review process was initiated during the study, after data collection had begun. Following review of the study documentation by the institutional health research orientation service, the research team was subsequently informed on 23 May 2025 that ethics committee review was not required for this study.

Participation was voluntary. Participants were informed of the study objectives and data confidentiality, consent was obtained before each interview, and data were anonymised for analysis and reporting using numbered identifiers.

## 3 Results

Ten occupational physicians participated in the study. Their main characteristics are presented in Table 1. Participants varied in age, professional seniority, career pathway and occupational sectors, and most had substantial experience in occupational health. Analysis identified four main domains: perceptions of the reform and changes in professional role; barriers and perceived facilitators to practice change; training and support; and health promotion practices.

**Table 1:** Characteristics of participating occupational physicians (n = 10).

Participant	Age group (birth cohort)	Years practising occupational medicine	Training pathway	Main practice sectors
Dr1	1965–70	10–20	Occupational medicine residency	Retail, industry, health and social care
Dr2	1990–95	<10	Occupational medicine residency	Retail, industry, food industry
Dr3	1960–70	>20	Occupational medicine residency	Food services, personal care services, nursing homes
Dr4	1960–65	<10	Career change	Retail, banking sector, higher education
Dr5	1965–70	>20	Occupational medicine residency	Construction sector, public administration
Dr6	1960–65	>20	Career change	Public administration, industry, construction sector, automotive services
Dr7	1970–75	>20	Occupational medicine residency	Public administration, public hospitals
Dr8	1960–65	>20	Occupational medicine residency	Public administration, industry, retail
Dr9	1960–65	>20	Career change	Industry, food services, retail, construction sector
Dr10	1965–70	>20	Career change	Food industry, food services, public administration

Note: Participant identifiers correspond to codes used in Results.

Participants expressed contrasting perceptions of the expansion of public health missions. Several considered occupational physicians' contribution to public health relevant, particularly when linked to workers’ health, but questioned the place of these missions in relation to responsibilities specific to occupational health. Prevention of occupational risks and support for continued employment were generally considered priorities.

Dr8 explained:

“It is very relevant, but it should not be a priority. Employers need to understand that we are here first and foremost to provide occupational health. But it is an added value. Otherwise, we risk moving away from our role.” (Dr8)

Dr3 similarly related this prioritisation to workforce constraints:

“Given the shortage of physicians, priorities are necessary: continued employment, occupational risks, safety, for example addictions. […] All other professions can contribute to public health. The role of specialist in occupational risks [remains] the priority.” (Dr3)

Reported barriers were mainly organisational. Limited time was recurrently associated with workload, staffing constraints and consultations involving complex or urgent occupational-health issues, leaving limited opportunities for additional health promotion activities.

Dr10 stated:

“Time. We are limited by consultation time; in 20–30 minutes, we don't have time. These are often consultations involving a problem, so we don't even address it. Sometimes their problems are related to public health, so it can be relevant.” (Dr10)

Dr9 similarly linked resources, workload and prioritisation:

“Lack of human resources, time, workload. If we conduct more consultations, we will put public health aside rather than occupational health, which is our core activity.” (Dr9)

Other reported barriers included difficulties coordinating with other healthcare professionals, the absence of clearly identified networks or contacts, and organisational difficulties in supporting change. Potential facilitators were more often described as desirable conditions than as measures participants had directly experienced. These included dedicated time, additional medical and nursing staff, multidisciplinary involvement, training resources and improved coordination with external stakeholders. A few participants nevertheless reported existing multidisciplinary support or specifically allocated resources.

Most participants reported little specific support for integrating the public health missions introduced or reinforced by the reform. Implementation was often described as relying on internal service organisation and individual professional initiative.

Dr5 summarised:

“They change the law and then say, ‘organise yourselves’. No particular support. What would be important is to have someone trained within the service.” (Dr5)

Specific training on public health missions was also rarely reported. When training had occurred, it mainly concerned other areas of occupational health. Participants expressed needs for updated information, clearer objectives, practical tools and support for translating new missions into action.

Dr6 explained:

“There isn't any. It requires real know-how that we don't have. We would need to be trained, to have objectives and to put tools in place.” (Dr6)

No single preferred training format emerged; participants mainly emphasised the need for updated content directly applicable to practice.

Several participants reported little or no change in activity directly attributable to the reform. Some missions now explicitly included in the regulatory framework were described as having already formed part of their practice, generally within individual consultations or usual occupational health activities rather than specific programmes initiated following the reform.

Dr1 described this continuity:

“Vaccination, but that was already in place before the law. We also carry out awareness activities regarding disability, but that was already the case before the law. Screening was also already in place before the law of 2 August 2021.” (Dr1)

Health promotion practices varied between physicians and according to the situations encountered. Physical activity and smoking were addressed during consultations, sometimes regularly but often according to the clinical or occupational context.

Dr7 explained:

“Physical activity, I systematically address it, and smoking too. During periodic consultations, I systematically ask about it. The problem is that these do not represent the majority of the consultations we conduct.” (Dr7)

Physical activity was notably discussed in relation to musculoskeletal disorders, cardiovascular risk factors or psychological difficulties, and several participants indicated that these practices predated the reform. Vaccination and screening practices also varied. Several participants prioritised interventions related to occupational risks, whereas interventions targeting the general population were sometimes considered more appropriately addressed by general practitioners. Others occasionally intervened beyond this scope, particularly when access to healthcare was difficult. Structured campaigns were rarely reported.

Disability-related activities were generally described through individual situations, including information about disability recognition, workplace adjustments, support for continued employment or referral to specialised partners. Some participants also reported employer-focused or collective activities, although implementation varied according to context. Formal evaluation appeared limited: several reported no specific indicators of effectiveness, while others relied mainly on individual follow-up or indirect information from their activity.

## 4 Discussion

This study found contrasting responses to the expansion of public health missions among the occupational physicians interviewed. While several considered occupational health involvement in broader public health objectives relevant, few reported changes in practice directly attributable to the 2021 reform. Vaccination, screening, physical activity and disability-related activities were often described as having predated it. The reform may therefore have formalised or increased the visibility of some pre-existing practices rather than immediately transforming practice. This interpretation remains cautious, as the study explored retrospective perceptions and did not objectively measure changes before and after the reform.

Organisational conditions appeared central to the translation of these expectations into practice. Limited time, workload and staffing constraints were the main barriers reported, consistent with research showing that implementation depends not only on professional acceptability but also on working conditions and available resources. [1], [2], [3], [4] Dedicated time, additional human resources and multidisciplinary support were generally described as desirable conditions rather than systematically available facilitators. This suggests that expanding regulatory missions without corresponding organisational capacity may limit their practical implementation.

The findings also highlight a tension concerning the boundaries of the occupational physician's role. Reservations about the reform did not necessarily reflect opposition to health promotion: participants reported addressing physical activity, addictions, disability and vaccination, particularly when these could be related to work or workers' health. However, prevention of occupational risks and support for continued employment were generally considered priorities, while some interventions primarily concerning general population health were perceived as lying closer to the boundaries of the specialty. Previous studies have also shown variation in how occupational health functions and competencies are prioritised, including the place of health promotion within occupational medicine. [7], [8], [9] In this study, these tensions coexisted with reported health promotion practices, suggesting negotiation of professional boundaries rather than rejection of the expanded missions.

Participants also reported limited training and support specifically addressing the public health dimensions of the reform. They expressed needs for updated information, clearer objectives and practical tools, although no single preferred training format emerged. Training can contribute to professional competencies and practice, but educational interventions alone may not overcome persistent organisational barriers. [1], [2], [5], [6], [7] Support for expanded missions may therefore require both practical training resources and organisational conditions allowing them to be implemented.

### 4.1 Strengths and limitations

Several limitations should be considered. The sample was small, and nine of the ten participants worked within the same occupational health service. Although the service comprised geographically distinct centres and participants varied in professional background and occupational sectors, this organisational concentration limits the diversity of contexts represented and the transferability of the findings to occupational physicians in France as a whole. Recruitment was mainly based on voluntary participation following a collective invitation, creating potential self-selection bias.

Participant characteristics were collected to describe and contextualise the sample rather than to support subgroup comparisons. Given the small sample size and the exploratory qualitative aim of the study, we did not undertake a systematic analysis of variations according to age, professional seniority or practice setting.

The interviews were not audio-recorded. Responses were typed in real time, with participants asked to repeat or clarify statements when necessary. This cannot ensure the same completeness and precision as audio recording followed by full transcription and may have resulted in loss of nuance. Notes were not subsequently checked by participants, and no separate field notes were kept.

Initial coding was conducted by a single researcher, and the full dataset was not independently double-coded. However, repeated detailed review of the coding table with the research supervisor allowed interpretations to be challenged and refined, categories to be further explored and some classifications to be reconsidered. This could not eliminate the subjectivity inherent in qualitative analysis.

All interviews were conducted by the first author, then an occupational medicine resident in his fifth semester of training undertaking his first qualitative study. Nine participants worked in his placement service, and pre-existing relationships with participants were professional in nature and varied in degree. Although an open, non-evaluative interviewing approach was sought, the researcher's position, professional relationships and limited qualitative research experience may have influenced data collection and interpretation.

These limitations mean that the findings should be understood as a contextualised exploration of the perceptions and self-reported practices of the physicians interviewed rather than a representative description of occupational physicians as a whole. Nevertheless, semi-structured interviews enabled detailed exploration of converging and contrasting perspectives across participants with varied professional seniority, career pathways and occupational sectors. Concurrent analysis and repeated review with the research supervisor also contributed to refining the analysis.

Overall, the findings suggest that regulatory expansion of professional missions does not necessarily translate into immediate changes in practice. Among the physicians interviewed, implementation was described in relation to organisational constraints and perceived boundaries of the occupational physician's role, while several health promotion activities were reported to have predated the reform. Research involving more diverse organisational settings could examine whether the reform has contributed not only to new practices but also to greater formalisation, coordination or evaluation of pre-existing health promotion activities.

## Authors’ contributions

Conceptualisation: BC, EF, MB; Methodology: BD, BC, EF, MB; Writing - original draft preparation: BD, MB; Writing - review & editing: BD, BC, EF, MB. Each author has been sufficiently involved in this work to take public responsibility for appropriate portions of its content.

## Ethical statement

The institutional ethics review process was initiated during the study, after data collection had begun. Following review of the study documentation by the institutional health research orientation service, the research team was subsequently informed on 23 May 2025 that ethics committee review was not required for this study. Participation was voluntary, consent was obtained from all participants before each interview, and the data were anonymised.

## Funding

This research received no specific grant from any funding agency in the public, commercial, or not-for-profit sectors.

## Declaration of competing interest

The authors declare that they have no known competing financial interests or personal relationships that could have appeared to influence the work reported in this paper.
