# Supplementary material for: Occupational physicians’ perceptions of professional role change following health policy reform in France: A qualitative study
Source: Public Health Pract (Oxf). 2026 Oct 10;12:100872. doi: 10.1016/j.puhip.2026.100872 (PMC13682051; doi:10.1016/j.puhip.2026.100872)
Supplement: Supplementary file 1 [file mmc1.docx]

**Supplementary Material 2
Coding Framework**

*Occupational physicians’ perceptions of professional role change following health policy reform in France: a qualitative study*

This summary coding framework was derived from the working coding table used during analysis. English labels were harmonised for clarity while preserving the analytical structure and meaning of the original coding. Main analytical domains were linked to the study objectives and interview-guide domains, while subthemes and ideas were refined or added during analysis.

*Note: Example coded extracts are presented as short units of meaning reflecting the coding process. Longer contextualised quotations are provided in the Results section of the manuscript. Extracts were translated from French into English for this supplementary material and are illustrative rather than exhaustive.*

| **Analytical domain** | **Subtheme** | **Code / idea** | **Operational description** | **Example coded extract** |
| --- | --- | --- | --- | --- |
| Perceptions of the reform and professional role change | Perceived relevance of public health objectives | Recognition of relevance | Public health promotion was viewed by some participants as a legitimate or useful component of occupational health practice. | “Yes, it is entirely relevant; it is a key issue.” (Dr 2) |
| Perceptions of the reform and professional role change | Perceived relevance of public health objectives | Legislator perceived as distant from field realities | Some reservations reflected a perceived gap between regulatory expectations and the realities of occupational health practice. | “We have the feeling that legal experts do not look at the profession through the same lens; legislative bureaucracy has no idea of field realities.” (Dr 1) |
| Perceptions of the reform and professional role change | Perceived relevance of public health objectives | Relevance conditional on a link with work | Some participants considered public health activities relevant mainly when they could be linked to occupational exposures, risks or work situations. | “It depends what it is. It can be relevant if there is a risk to the worker, for example silica and smoking.” (Dr 6) |
| Perceptions of the reform and professional role change | Professional role boundaries | Priority given to occupational health | Public health activities could be accepted as complementary while remaining secondary to occupational health missions, particularly occupational risk prevention. | “I find it very relevant, but it must not be a priority.” (Dr 8) |
| Perceptions of the reform and professional role change | Professional role boundaries | Risk of shifting responsibility towards workers | Some participants feared that health-promotion messages focused on individual behaviours could shift attention away from employers’ responsibility for occupational risks. | “I perceive a risk of introducing workers’ responsibility. Basically, if they are unwell, it becomes their fault because they do not exercise or something similar.” (Dr 3) |
| Perceptions of the reform and professional role change | Perceived change following reform | Limited change directly attributed to the 2021 reform | Participants often reported little change in their public-health-related practice after the reform. | “There was a real before-and-after with the creation of multidisciplinary teams, but we felt less change after the law of 2 August 2021.” (Dr 3) |
| Perceptions of the reform and professional role change | Perceived change following reform | Changes attributed more to other dimensions of the reform | When change was reported after the reform, some participants attributed it more to other dimensions of the reform than to health promotion. | “Yes, there is change, but more on the occupational-health side than on public health.” (Dr 4) |
| Barriers and perceived facilitators to practice change | Potential facilitators / perceived enabling conditions | Dedicated time | Protected or specifically dedicated time was described as a condition that could facilitate additional health-promotion activities. | “Dedicated time would be needed, but that would be more within the remit of occupational health nurses.” (Dr 2) |
| Barriers and perceived facilitators to practice change | Potential facilitators / perceived enabling conditions | Additional staffing and multidisciplinary support | Additional staff and support from the multidisciplinary team were described as potential resources for developing public health activities. | “If we were more numerous, perhaps we would have more time to do public health.” (Dr 7) |
| Barriers and perceived facilitators to practice change | Potential facilitators / perceived enabling conditions | Training and skills development | Access to appropriate training was described as a condition that could strengthen perceived competence and facilitate the integration of new practices. | “From a theoretical point of view, perhaps we would need training.” (Dr 7) |
| Barriers and perceived facilitators to practice change | Reported barriers | Lack of time | Limited consultation and organisational time constrained the addition of health-promotion activities. | “We do not have time.” (Dr 7) |
| Barriers and perceived facilitators to practice change | Reported barriers | Staffing constraints | Shortages of occupational health professionals were described as limiting the feasibility of additional missions. | “We lack staff.” (Dr 3) |
| Barriers and perceived facilitators to practice change | Reported barriers | Workload and need to prioritise | Workload and competing missions led some participants to prioritise activities, particularly those directly related to occupational health. | “We cannot do everything; that was already the case before the reform and it is still the case now.” (Dr 5) |
| Barriers and perceived facilitators to practice change | Reported barriers | Perceived insufficient competence in some public health areas | Some participants reported feeling insufficiently up to date or skilled in certain public health topics. | “It requires real know-how that we do not have.” (Dr 6) |
| Barriers and perceived facilitators to practice change | Limits of follow-up and evaluation | Few formal indicators | Participants reported few formal indicators for evaluating health-promotion activities, limiting their visibility and assessment of their effects. | “We do not have clear indicators apart from feedback from workers and employers and observation of turnover.” (Dr 1) |
| Training and support | Support needs | Training of the multidisciplinary team | Some participants considered developing public health skills within the multidisciplinary team as a possible way to support the new missions. | “It would be useful to have someone trained for this in each service.” (Dr 4) |
| Training and support | Support needs | Links with external professionals and networks | Participants described a need for clearer referral pathways, partnerships and information exchange with other health and public health professionals. | “It requires a lot of energy and links with external partners to establish a network that does not currently exist.” (Dr 6) |
| Training and support | Support needs | Clearer objectives, limits and practical tools | Participants requested more explicit expectations and practical guidance for implementing the new missions. | “We need more precise content regarding the new missions assigned.” (Dr 1) |
| Training and support | Perceived institutional support | Little specific support perceived | Several participants reported perceiving little or no specific institutional support for implementing the public health dimension of the reform. | “I do not have the impression that there was any support.” (Dr 2) |
| Training and support | Perceived institutional support | Mismatch between additional missions and available resources | Some participants perceived the reform as adding expectations without sufficiently accounting for existing organisational constraints. | “The 2021 law adds missions to services that are already understaffed.” (Dr 9) |
| Training and support | Training | Few training activities specifically related to the new public health missions reported | Training undertaken or offered after the reform was described by several participants as mainly focused on occupational health topics rather than specifically on the new public health missions. | “All the training offered is related to occupational health.” (Dr 10) |
| Training and support | Training | Varied training formats and directly applicable content | Preferences regarding training formats varied; some participants particularly valued content that could be readily applied in practice. | “Theoretical training with simple keys that are easy to put into practice during consultations.” (Dr 2) |
| Training and support | Training | Topic-specific updating or training needs | Updating or training needs were expressed for some health-promotion topics, including physical activity and nutrition. | “We would need updates and refreshed knowledge to provide recent information on what is being done in terms of sport and diet.” (Dr 7) |
| Health promotion practices | Overall reported practices | Diversity of reported health-promotion practices | Participants reported various individual or collective practices that could fall within health promotion, including addiction prevention, disability, physical activity, vaccination and screening. | “It is true that I have already carried out addiction-awareness activities.” (Dr 3) |
| Health promotion practices | Overall reported practices | Practices adapted to context and perceived competence | Health-promotion topics were addressed variably according to consultation context, identified risks, available time and perceived competence. | “During periodic visits I can discuss physical activity or smoking, but it is not the majority of visits.” (Dr 7) |
| Health promotion practices | Overall reported practices | Practices predating the reform | Several participants reported that activities highlighted by the reform were already part of their practice before 2021. | “The law did not change our discourse; when we identified a public health risk, we already addressed it before the law.” (Dr 3) |
| Health promotion practices | Vaccination and screening | Anchoring in occupational risks | Vaccination and screening were notably addressed when linked to occupational exposure or risk; their integration into a broader public health approach appeared more variable. | “I deal with mandatory vaccinations related to the job.” (Dr 6) |
| Health promotion practices | Physical activity | Physical activity addressed contextually | Physical activity was addressed by several participants, particularly in relation to occupational constraints, musculoskeletal risks or specific health situations. | “It is a topic I discuss more and more given the increase in low-back disorders.” (Dr 5) |
| Health promotion practices | Disability at work | Practice already integrated into occupational health missions | Disability was described as already embedded in occupational health practice, particularly through work accommodation and compensation, and was not necessarily perceived as a new mission resulting from the reform. | “I do not do much awareness-raising; I mainly do work accommodation.” (Dr 5) |
