# Supplementary material for: Occupational physicians’ perceptions of professional role change following health policy reform in France: A qualitative study
Source: Public Health Pract (Oxf). 2026 Oct 10;12:100872. doi: 10.1016/j.puhip.2026.100872 (PMC13682051; doi:10.1016/j.puhip.2026.100872)
Supplement: Supplementary file 2 [file mmc2.docx]

**Supplementary Material
Interview Guide**

*Occupational physicians’ perceptions of professional role change following health policy reform in France: a qualitative study*

**Presentation and reminder of the research project**

- To describe and analyse occupational physicians’ perceptions of the reform of 2 August 2021, particularly regarding the introduction of workplace health promotion activities;
- To identify facilitators and barriers to changes in professional competencies and practices within this new framework;
- To assess the extent to which public health principles are reflected in occupational physicians’ field practices;
- To explore the training and support needs expressed by these professionals in relation to the new missions assigned to them.

**Brief reminder of the legislative framework**

**Before the Law of 2 August 2021:** “Occupational health services have the exclusive mission of preventing any deterioration in workers’ health resulting from their work.”

- Occupational health actions to preserve workers’ health throughout their working lives;
- Advice regarding occupational risks, drug or alcohol use in the workplace, sexual or psychological harassment, prevention of occupational disengagement, and support for continued employment;
- Monitoring workers’ health according to identified risks;
- Traceability of exposures and health surveillance.

**After the Law of 2 August 2021:** “Occupational health services have the primary mission of preventing any deterioration in workers’ health resulting from their work. They contribute to achieving public health objectives in order to preserve, throughout working life, a state of health compatible with continued employment.”

- Occupational health actions to preserve workers’ health throughout their working lives;
- Assessment and prevention of occupational risks;
- Advice regarding occupational risks, quality of working life, telework, drug or alcohol use in the workplace, sexual or psychological harassment, prevention of occupational disengagement, and support for continued employment;
- Support during major organisational changes within the company;
- Monitoring workers’ health according to identified risks;
- Traceability of exposures and health surveillance;
- Workplace health promotion (vaccination and screening, raising awareness of physical activity, disability and work) within the framework of the national health strategy set out in the Public Health Code.

*Participants were reassured that the responses collected would remain confidential and that the interview was not intended as an audit of practice, but rather as an assessment of current practices.*

**Participant characteristics**

**1)** Sex: (F / M)

**2)** Age: (year of birth)

**3)** Seniority as an occupational physician: (number of years)

**4)** Seniority within the occupational health service: (number of years)

**5)** Initial pathway (occupational medicine residency or former collaborating physician/career change): Did you have another medical specialty before becoming an occupational physician? If so, which one?

**6)** Number of companies followed?

**7)** Types of companies in your portfolio (private/public, size): (private / public / mixed)

**8)** Number of employees/workers followed:

**9)** Effective workplace activity time per week: Does your workplace activity time effectively represent one third of your working time?

**10)** During this time outside medical visits, what activities do you carry out? Please list them.

**Themes and questions**

**Perceptions of changes in practice**

**11)** Do you consider it relevant for an occupational physician to carry out public health missions?

**12)** In your opinion, what are the essential competencies (technical knowledge, interpersonal skills, team management, etc.) for an occupational physician in the current context of increasing missions assigned by the legislator? / In the current context of a shortage of occupational physicians and an expansion of their scope of action, what competencies do you think occupational physicians need?

**13)** Since the Law of 2 August 2021, have you perceived any change in your professional scope and missions? Why? / How has the 2022 reform changed your activity?

**14)** How do you assess the impact of these changes on your daily practice as an occupational physician? / Can you provide examples of changes in your daily practice since the Law of 2 August 2021?

**Barriers and factors facilitating changes in competencies and practices**

**15)** What factors have facilitated any changes in your practices? What factors facilitating changes in occupational health practices can you identify?

**16)** What are the main difficulties you encounter in implementing these activities? In your opinion, what are the barriers to changes in occupational health practices in the context of the Law of 2 August 2021?

**Training and support**

**17)** What needs do you identify in terms of resources or support to implement these new missions? Considering the realities and difficulties encountered in practice, what do you need to carry out the missions assigned to occupational physicians?

**18)** How do you perceive the support provided to occupational physicians in the context of this reform? Have you received any support to implement the new missions assigned to occupational physicians?

**19)** Have you undertaken specific training since 2022 to adapt to the new missions and responsibilities of occupational physicians? If so, which training? What types of training have you undertaken in relation to the 2022 reform?

**20)** In your opinion, what type of training is most effective for developing new competencies as an occupational physician following the reform? / What types of training seem appropriate in relation to the changes introduced by the 2022 reform?

**21)** Do you have any suggestions or recommendations for improving support for occupational physicians in this context of change? / Do you have any recommendations regarding the resources allocated to these new missions? If so, which ones?

**22)** On which subjects or themes would you like to receive more training or support? / Are there specific topics among those newly introduced by the 2022 reform for which you feel you need additional support?

**Practices**

**23)** What concrete actions have you implemented regarding workplace health promotion? More generally, what workplace health promotion activities do you carry out?

**24)** How do you measure the effectiveness of your workplace health promotion activities? / What indicators do you use to assess the effectiveness of these activities?

**25)** How would you describe your practices regarding vaccination and screening campaigns among workers? / Do you conduct vaccination and screening campaigns among the employees you follow? If so, how?

**26)** Do you raise workers’ awareness of the benefits of physical activity in the occupational context? If so, how? / Do you conduct activities to raise awareness of the benefits of physical activity in the occupational context? If so, which ones?

**27)** What information and awareness-raising activities do you provide regarding disability in the workplace? / Do you conduct awareness-raising activities regarding disability in the workplace? If so, which ones?

*Thank you for your participation.*
